# Cell-Specific Cre Strains For Genetic Manipulation in Salivary Glands

**DOI:** 10.1371/journal.pone.0146711

**Published:** 2016-01-11

**Authors:** Eri O. Maruyama, Marit H. Aure, Xiaoling Xie, Yvonne Myal, Lin Gan, Catherine E. Ovitt

**Affiliations:** 1 Center for Oral Biology; Department of Biomedical Genetics, University of Rochester Medical Center, Rochester, New York 14642, United States of America; 2 Department of Ophthalmology; Department of Neurobiology and Anatomy, University of Rochester Medical Center, Rochester, New York, United States of America; 3 Department of Pathology; Department of Physiology and Pathophysiology, Faculty of Health Sciences, University of Manitoba, Winnipeg, Canada; National Institute of Dental and Craniofacial Research, UNITED STATES

## Abstract

The secretory acinar cells of the salivary gland are essential for saliva secretion, but are also the cell type preferentially lost following radiation treatment for head and neck cancer. The source of replacement acinar cells is currently a matter of debate. There is evidence for the presence of adult stem cells located within specific ductal regions of the salivary glands, but our laboratory recently demonstrated that differentiated acinar cells are maintained without significant stem cell contribution. To enable further investigation of salivary gland cell lineages and their origins, we generated three cell-specific Cre driver mouse strains. For genetic manipulation in acinar cells, an inducible Cre recombinase (Cre-ER) was targeted to the prolactin-induced protein (*Pip*) gene locus. Targeting of the *Dcpp1* gene, encoding demilune cell and parotid protein, labels intercalated duct cells, a putative site of salivary gland stem cells, and serous demilune cells of the sublingual gland. Duct cell-specific Cre expression was attempted by targeting the inducible Cre to the *Tcfcp2l1* gene locus. Using the R26^Tomato Red^ reporter mouse, we demonstrate that these strains direct inducible, cell-specific expression. Genetic tracing of acinar cells using Pip^GCE^ supports the recent finding that differentiated acinar cells clonally expand. Moreover, tracing of intercalated duct cells expressing Dcpp^GCE^ confirms evidence of duct cell proliferation, but further analysis is required to establish that renewal of secretory acinar cells is dependent on stem cells within these ducts.

## Introduction

**Fig 1:**
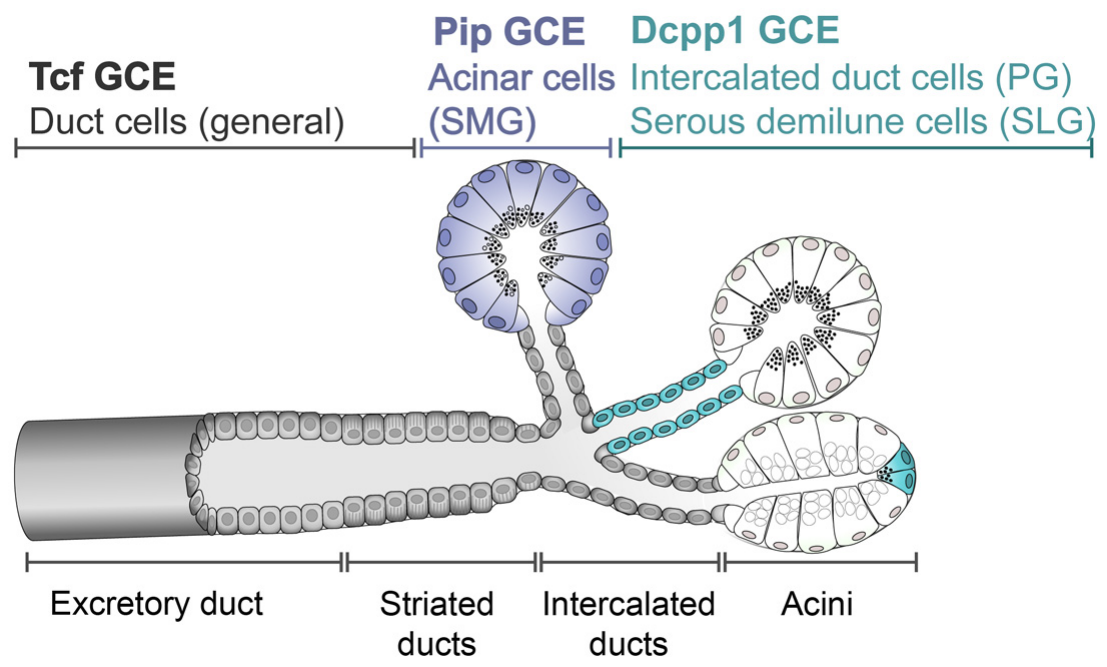
Schematic diagram of general salivary gland structure. Secretory acinar cells are arranged in clusters, known as acini, which produce primary saliva. The smallest intercalated ducts conduct saliva from the acini to the striated, and excretory ducts. Sites of inducible Cre drivers are indicated, color-coded for each strain. *Dcpp1*, gene encoding demilune cell and parotid protein; *Pip*, gene encoding prolactin-inducible protein; *Tcf*, gene encoding Tcfcp2l1 transcription factor.

The salivary glands are responsible for the secretion of saliva, which is essential for oral health. The major cellular component of the salivary glands is the secretory acinar cells (reviewed in [1]), which are arranged in clusters. The acinar cells secrete primary saliva into the small, intercalated ducts, which are linked to striated ducts. Eventually, the saliva is conducted through the ductal tree to the large excretory ducts, which empty into the oral cavity (Fig 1). A decrease in saliva secretion leads to the condition known as xerostomia and results in debilitating health problems. Saliva secretion is severely reduced by radiation therapy to treat head and neck cancers, and as a consequence of the autoimmune disease, known as Sjögren’s syndrome. In both cases, the underlying cause is an irreversible loss of the acinar cells [2]. Thus, repair or regeneration of the salivary glands is primarily concentrated on replacement of the secretory cells. Current strategies to accomplish this are focused on the use of putative adult stem cells [3–5]. The prevailing view is that stem cells are localized to the small intercalated, and large excretory ducts in the salivary gland (reviewed in [6]) (see Fig 1). However, evidence of their differentiation into acinar cells has not yet been directly demonstrated. Furthermore, in a study to directly determine the source of newly generated acinar cells, we found that there is little stem cell contribution to acinar cell renewal in adult salivary glands [7].

In order to further investigate the role of each cell type in salivary gland homeostasis, we have generated three cell-specific inducible Cre recombinase mouse strains. The prolactin-induced protein (Pip) is a secretory glycoprotein produced by serous cells of the mouse and human salivary glands [8–10]. The *Pip* gene locus was targeted to generate a Cre-driver active in acinar cells. To label intercalated duct cells, the presumptive site of stem cells in the parotid gland, the demilune cell and parotid protein gene (*Dcpp1)* [11], one of three linked Dcpp-related genes on mouse chromosome 17 [12], was targeted. Dcpp1 is also a marker of serous demilune cells in the sublingual gland [13]. Each acinus of the sublingual gland is comprised of mucous-secreting cells and one or two serous cells, distinguished by their expression of Dcpp1 (see Fig 1) [11, 14, 15], and of Sox2, a stem cell marker [7, 16]. A third Cre-driver strain was generated to label duct cells by targeting the *Tcfcp2l1* gene locus, which encodes a transcription factor specifically expressed in duct cells of the developing kidney and all three major salivary glands [17, 18]. Although this line does show Cre activation in duct cells, ectopic expression in acinar cells may limit its usefulness in lineage tracing studies.

## Results

### Pip^GCE^ labels acinar cells in the submandibular gland

**Fig 2:**
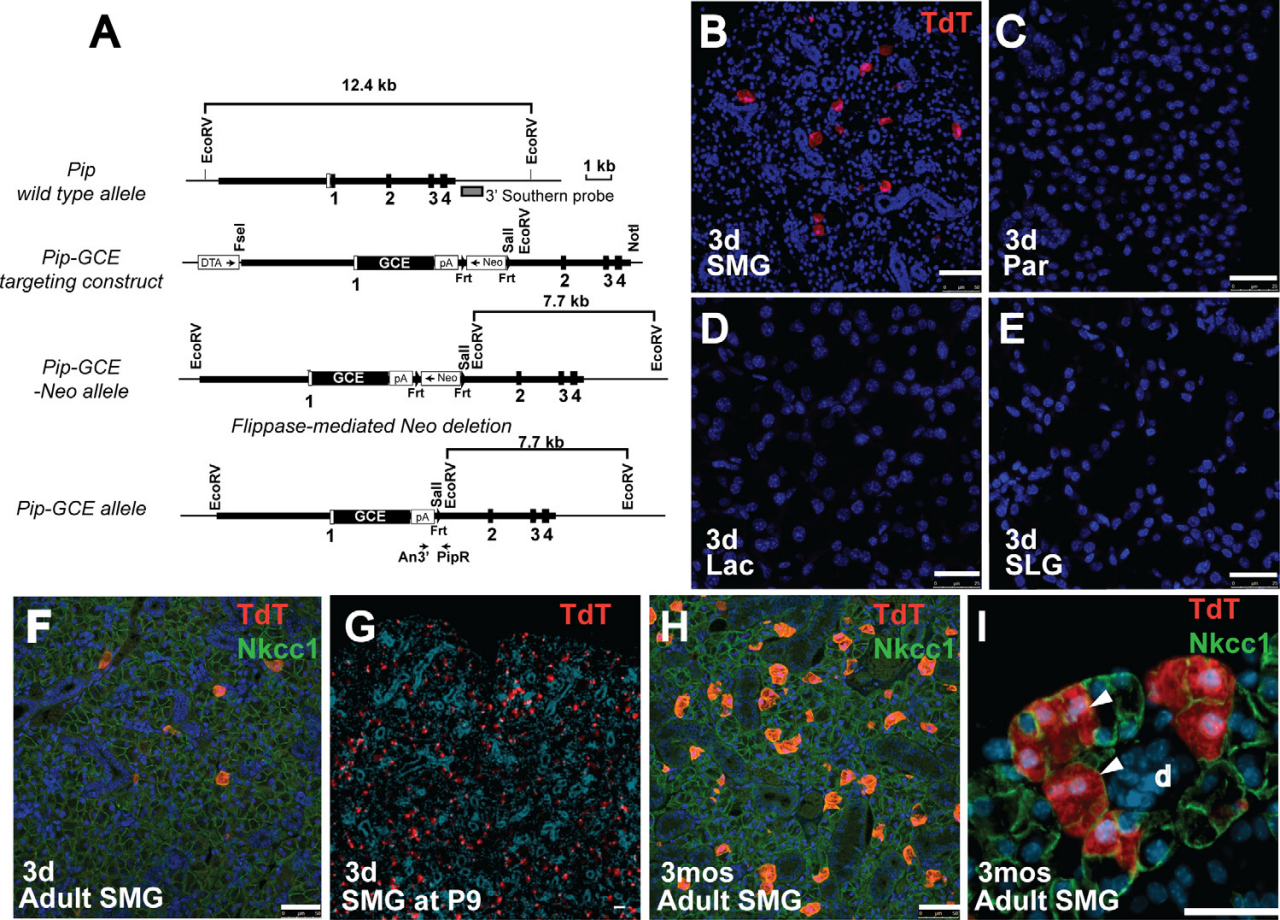
Characterization of *Pip*^*GCE*^ knock-in allele. **(A)** Generation of *Pip*^*GCE*^ knock-in mice. *Pip* genomic structure and restriction map is shown at the top. White box represents the non-coding exon sequences and filled boxes, the coding sequences. Thick bars show the sequences used to generate the homologous arms in the targeting vector. Gray box represents 3’ external probe used for Southern blotting. Arrows indicate positions of genotyping PCR primers (An3’ and PipR). **(B-E)** Analysis of Cre expression in mice after 3 days of tamoxifen treatment, followed by a 3-day chase. **(B)** Frozen sections were prepared from submandibular gland (SMG); activation of Cre results in expression of Tomato red reporter (TdT) (red); Scale bar = 50 *μ*m. No Cre activity is detected in **(C)** parotid (Par), **(D)** lacrimal gland (Lac) or **(E)** sublingual gland (SLG). Nuclei are stained with DAPI (blue). Scale bars = 25*μ*m. **(F)** Section from *Pip*^*GCE/+*^*;R26* ^*TdT/+*^ SMG at 3 days after tamoxifen treatment. Single labeled acinar cells (red) co-localize with antibody to Nkcc1 (green). Scale bar = 50*μ*m **(G)** Section from *Pip*^*GCE/+*^*;R26* ^*TdT/+*^ SMG at P9, isolated 3 days after tamoxifen administration. Positively labeled acinar cells are red. Nuclei are stained with DAPI. Scale bar = 25*μ*m **(H)** Section from *Pip*^*GCE/+*^*;R26* ^*TdT/+*^ SMG at 3 months after tamoxifen treatment, co-stained with antibody to Nkcc1 (green) to label acinar cells. Labeled acinar cells have expanded to clones (red). Scale bar = 50*μ*m **(I)** Section from *Pip*^*GCE/+*^*;R26* ^*TdT/+*^ SMG after 3 month chase shows expansion of labeled acinar cells into clones (arrowheads). *3d*, 3 days chase; *3mos*, 3 month chase; *d*, duct; Scale bar = 50 *μ*m.

The *Pip* gene (gene ID 18716) was targeted by homologous recombination with a fusion cassette encoding GFP and CreER^T2^ (GCE) [19] to remove the coding sequences from Exon 1 and place the GCE cassette under the control of *Pip* regulatory sequences (Fig 2A). To determine the pattern of GCE expression, *Pip*^*GCE/+*^ heterozygote males were crossed with females from the *Gt(ROSA)26Sor*^*tm9(CAG-tdTomato)Hze*^*/J* reporter strain, hereafter referred to as *R26*^*TdT*^. Double heterozygous *Pip*^*GCE/+*^ */ R26*^*TdT/+*^ animals (3 weeks old) were administered tamoxifen by gavage for 3 consecutive days. Tissues were harvested after a 3-day chase, and frozen sections were examined for RFP fluorescence. Labeled cells were detected specifically in the submandibular gland (SMG) (Fig 2B). In contrast to the endogenous expression of Pip in parotid, sublingual and lacrimal glands (S1 Fig)[20], there was no evidence of Cre activation in these tissues (Fig 2C–2E). To ascertain the cell type expressing Pip^GCE^, sections of SMG were co-stained with antibody to Nkcc1, which labels acinar cell membranes (Fig 2F). All tomato red-positive cells are co-localized with Nkcc1, indicating that they are acinar cells. Pip^GCE^ expression was not detected in duct cells. No expression of R26^TdT^ was detected in the absence of tamoxifen (data not shown).

The expression of *Pip* is initiated by embryonic day 14 (E14), and marks proacinar cells in the developing SMG [21]. However, administration of tamoxifen to pregnant females on 2 consecutive days failed to induce Pip^GCE^ activity in embryonic SMG after a 2-day chase when analyzed at E15.5 or E17.5 (data not shown). In contrast, tamoxifen administered by gavage on postnatal days 4 (P4) through P6 labeled a large number of acinar cells in the SMG by P9 (Fig 2G), although not in parotid, lacrimal or sublingual glands (data not shown). Pip expression is limited to apocrine glands of the eye, ear canal, and reproductive organs [22]. In agreement, we detected no activation of Pip^GCE^ in kidney, lung, pancreas, prostate, or ovary (data not shown) following tamoxifen administration. The *Pip*^*GCE*^ allele therefore represents a specific Cre driver for genetic manipulation in the SMG.

We have recently reported that differentiated acinar cells in the adult salivary glands continue to proliferate, and are maintained through self-duplication [7]. As the *Pip*^*GCE*^ allele drives tightly controlled, inducible Cre expression in postnatal and adult SMG acinar cells, we examined whether this system can also be used to follow clonal expansion. Single acinar cells were genetically labeled in heterozygous *Pip*^*GCE*^*; R26*^*TdT/+*^ mice (3 weeks old) by administering tamoxifen for 3 consecutive days (Fig 2F). After a chase period of 3 months, labeled acinar cells are present in clusters, evidence of clonal expansion through self-duplication (Fig 2H and 2I), as described [7]. Thus, the *Pip*^*GCE*^ can be used to genetically label or modify an expanding population of secretory acinar cells in the SMG.

### Dcpp1^GCE^ labels sublingual serous demilune cells and parotid gland intercalated duct cells

**Fig 3:**
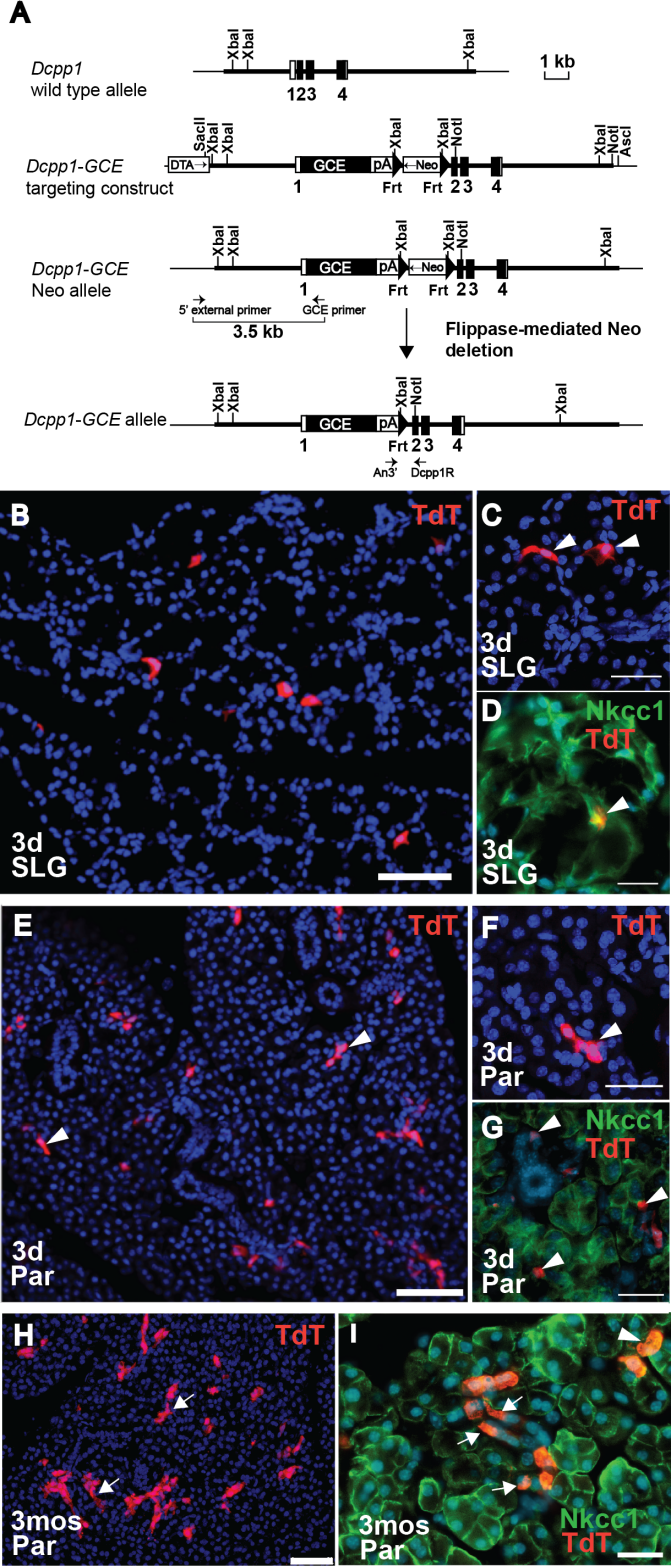
Characterization of *Dcpp1*^*GCE*^ knock-in allele. **(A)** Generation of *Dcpp1*^*GCE*^ knock-in mice. *Dcpp1* genomic structure and restriction map is shown at the top. White box represents the non-coding exon sequences and filled boxes, the coding sequences. Thick bars show the sequences used to generate the homologous arms in the targeting vector. Arrows indicate positions of external long-range PCR primers (5’ external primer and GCE primer) and internal primers (An3’ and Dcpp1R) used for genotyping. **(B)** Analysis of Cre expression in sublingual gland (SLG) of *Dcpp1*^*GCE/+*^*;R26* ^*TdT/+*^ mice after 3 days of tamoxifen treatment, followed by a 3-day chase. Activation of Cre results in expression of Tomato red reporter (TdT) (red). **(C)** Higher magnification of labeled SLG cells reveals the morphology of serous demilunes (arrowheads). **(D)** Antibody to Nkcc1 labels SLG acinar cell membranes, and co-localizes with TdT-labeled serous demilune cell (yellow; arrowhead). **(E)** Analysis of Cre expression in parotid gland (Par) of *Dcpp1*^*GCE/+*^*;R26* ^*TdT/+*^ mice after 3 days of tamoxifen treatment, followed by a 3-day chase. Activation of Cre results in expression of TdT (red) in small clusters of intercalated duct cells (arrowheads). **(F)** Higher magnification of TdT-labeled (red) intercalated duct cells (arrowhead). Nuclei are stained with DAPI (blue). **(G)** Antibody to Nkcc1 labels acinar cells (green). TdT-positive cells (red) do not co-localize with acinar cells, but are found within the smallest intercalated ducts (arrowheads). **(H)** Section from *Dcpp1*^*GCE/+*^*;R26* ^*TdT/+*^ parotid gland after 3 days of tamoxifen treatment, followed by a 3-month chase. TdT-positive cells (red) are clustered in duct-like structures (arrows). **(I)** At 3 months chase, TdT-labeled cells (red) derived from Dcpp1-expressing cells are clustered in intercalated ducts (arrows). Some Dcpp1-labeled cells may overlap with acinar cells labeled with antibody to Nkcc1 (green; arrowhead). Nuclei are stained with DAPI (blue). *3d*, 3 days chase; *3mos*, 3 month chase; Scale bars = 50*μ*m (B,E,H); = 25*μ*m (C,D,F,G); = 20*μ*m (I).

The GCE fusion cassette [19] was inserted into the *Dcpp1* gene (gene ID 13184) at the initiation site in Exon 2 through homologous recombination (Fig 3A). To assess the Cre expression pattern in this line, Dcpp1^GCE^ heterozygote males were mated with females from the *R26*^*TdT*^ reporter strain. Tamoxifen was administered by gavage to 3-week-old *Dcpp1*^*GCE/+*^*/ R26*^*TdT/+*^ mice on 3 consecutive days, and tissues were analyzed after a 3-day chase. In the sublingual gland (SLG), the tomato red reporter was activated in single cells (Fig 3B). Both cell morphology and co-staining with antibody to Nkcc1 indicate that the labeled cells are serous demilunes (Fig 3C and 3D), as expected based on endogenous Dcpp1 expression (see Fig 1 and S2 Fig). No expression was detected in mucous acinar cells. The availability of Dcpp1^GCE^ as a molecular tag for the serous acinar cell type will be useful for defining the specific role of serous demilune cells in the SLG.

Analysis of parotid glands after a 3-day chase also showed activation of Dcpp^GCE^ in a scattered cell population (Fig 3E). In the parotid gland, Dcpp1 is exclusively expressed in intercalated duct cells (see Fig 1 and S2 Fig) [11]. Higher magnification, as well as absence of co-localization with antibody to Nkcc1, was used to confirm that tomato red-positive cells are intercalated duct cells (Fig 3F and 3G). Thus, the *Dcpp1*^*GCE*^ allele faithfully recapitulates the expression pattern of the endogenous *Dcpp1* gene.

Intercalated ducts in the parotid gland have long been thought to be the site of salivary gland stem cells [23–26]. Short-term lineage tracing demonstrated progenitor activity in intercalated ducts [27], but lineage tracing from intercalated duct cells into acinar cells has not been reported. We used *Dcpp1*^*GCE*^*/ R26*^*TdT/+*^ mice to trace the intercalated duct cells over time. Tamoxifen was administered by gavage to 4-week-old mice for 3 consecutive days. After a 3-month chase, analysis of the parotid glands showed an increased number of labeled cells in each cluster (compare Fig 3H and 3E). Most of the TdT-positive cells remain within intercalated ducts (Fig 3I, arrows). However, there were some TdT-positive cells that co-localized with Nkcc1 antibody, suggesting that they may be acinar cells (Fig 3I, arrowheads). Given the widely held view that the intercalated ducts harbor stem cells, further characterization of these double-labeled cells will clearly be interesting, and underscores the potential utility of this Cre line. While the question of stem cells remains open, the low number of double-labeled acinar cells after a 3 month chase is in agreement with our recent conclusion [7] that the intercalated ducts do not make a significant contribution to replenishment of acinar cells under normal homeostatic conditions.

### Tcfcp2l1^GCE^ drives ectopic expression of Cre in salivary gland acinar as well as duct cells

**Fig 4:**
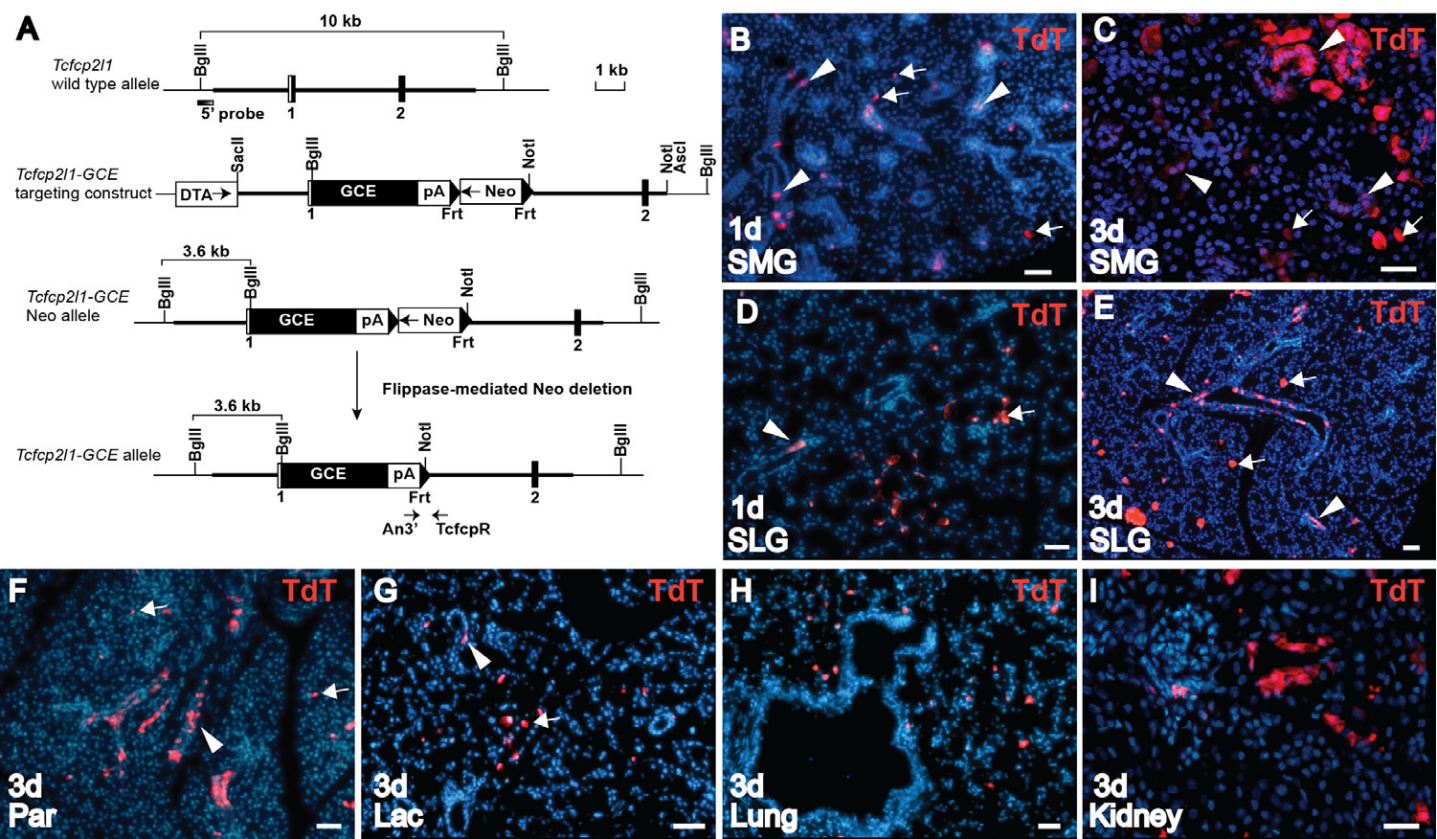
Characterization of *Tcf*^*GCE*^ knock-in allele. **(A)** Generation of *Tcf*^*GCE*^ knock-in mice. *Tcfcp2l1* genomic structure and restriction map is shown at the top. White box represents the non-coding exon sequences and filled boxes, the coding sequences. Thick bars show the sequences used to generate the homologous arms in the targeting vector. Shaded box represents 5’ external probe used for Southern blotting. Arrows indicate positions of genotyping PCR primers (An3’ and TcfcpR). **(B)** Analysis of Cre expression in *Tcf*^*GCE/+*^*;R26* ^*TdT/+*^ mice on frozen sections from submandibular gland (SMG) 1 day after a single tamoxifen gavage shows TdT-positive labeled duct cells (red; arrowheads) and acinar cells (red; arrows). Scale bar = 50*μ*m **(C)** Section of SMG after 3 days tamoxifen gavage shows TdT-positive Cre activity in ducts, granulated ducts of males (arrowheads) and acinar cells (arrows). Scale bar = 25*μ*m **(D)** Section of sublingual gland (SLG) after a single tamoxifen gavage at 1 day shows TdT-positive Cre activity in duct (arrowhead) and acinar cells (arrow). **(E)** SLG after 3 days tamoxifen gavage shows increased TdT-positive labeling of duct (arrowheads) and acinar cells (arrows). **(F)** Section of parotid (Par) after 3 days tamoxifen gavage shows TdT-positive labeling of both duct (arrowhead) and acinar cells (arrows). **(G)** Section of lacrimal (Lac) gland after 3 days tamoxifen gavage shows labeling of both duct (arrowhead) and acinar cells (arrow). **(H)** Section of lung after 3 days tamoxifen gavage shows Cre activity due to Tcf^GCE^ expression (red). **(I)** Section of kidney after 3 days tamoxifen gavage shows Cre activity due to Tcf^GCE^ expression (red). Nuclei stained with DAPI (blue). Scale bars = 50*μ*m; *1d*, 1 day chase; *3d*, 3 days chase.

The *Tcfcp2l1* gene (gene ID 81879) was targeted with the GCE fusion cassette at the initiation codon in Exon 1 (Fig 4A). The Cre expression pattern was investigated by mating *Tcf*^*GCE*^ with the *R26*^*TdT/+*^ reporter strain. Tamoxifen was administered by gavage to 3-week-old mice. After only one day and a single administration of tamoxifen, tomato red reporter expression was detected in the SMG, and SLG (Fig 4B and 4D). As expected, many labeled cells were in the ducts. However, Cre activation was also observed in acinar cells outside the ducts in both glands (Fig 4B and 4D; arrows). Tamoxifen treatment for 3 days resulted in significantly more labeled cells, showing that the labeling of acinar cells is dependent on tamoxifen induction (Fig 4C and 4E). When these cells were analyzed after 1 month, the labeled acinar cells had expanded into multicellular clones (data not shown), as would be expected based on our recent demonstration that acinar cells are maintained by self-duplication [7]. Tamoxifen activation of Tcf^GCE^ also induced reporter expression in both duct and acinar cells of the parotid glands (Fig 4F), and in the lacrimal glands, which produce tear secretions at the eye (Fig 4G).

*Tcfcp2l1* gene expression is initiated by E13.5 in development, and is required for the maturation of kidney and salivary gland duct cells [18]. To induce activation of Tcf^GCE^ in embryos, tamoxifen was administered intraperitoneally to pregnant females at E13.5, and sections were analyzed at E15.5. However, we found no evidence of Tomato red reporter expression in the embryonic salivary glands (data not shown). The pattern of Tcf^GCE^ expression was also analyzed in other tissues. In agreement with published expression patterns for the *Tcfcp2l1* gene [17, 18], tamoxifen did activate Tcf^GCE^ in single bronchial cells of the adult lung, and in ducts of the kidney cortex (Fig 4H and 4I).

We note that in all trials the use of the ROSA 26S^tm1Sor^ (R26^LacZ^) reporter yielded only limited detectable expression following tamoxifen induction (data not shown) and is not recommended with these GCE strains. Furthermore, although the GCE cassette comprises a GFP and CreER^T2^ in-frame fusion, we were unable to detect GFP fluorescence or GFP signal with antibody in any of the 3 strains (data not shown).

## Discussion

An understanding of salivary gland biology is essential for the development of therapies to treat salivary gland dysfunction, or to harness the glands for expression and secretion of heterologous proteins for therapeutic use. The parotid, submandibular and sublingual salivary glands bear morphological, developmental and molecular similarities, but also have distinct cellular compositions and secretory products. A better understanding of the biology of these glands will come only with the ability to manipulate or modify salivary gland gene expression.

We report the generation of three mouse strains driving expression of tamoxifen-inducible CreER^T2^ for genetic manipulation in the salivary glands. Knock-in targeting of the *Pip* gene drives Cre expression specifically in the secretory acinar cells of the SMG. Targeting of the *Dcpp1* gene yields Cre expression specifically in the serous demilune cells of the sublingual gland, as well as in parotid gland intercalated duct cells, the putative site of salivary gland stem cells. Targeting of the *Tcfcp2l1* gene directs Cre expression to duct cells in all three salivary gland types, as well as the lacrimal gland ducts, but also drives unexpected ectopic expression in acinar cells. Although selection of these genes was also based on their patterns of expression during embryonic salivary gland development, none of the three strains has thus far demonstrated evidence of prenatal activation of the GCE cassette.

In contrast to the widely held dogma that salivary gland homeostasis is dependent on stem cells, we have recently reported that maintenance of differentiated acinar cells in the postnatal salivary glands is accomplished through self-duplication [7]. Genetic cell labeling using the *Pip*^*GCE*^ allele also demonstrates that acinar cells continue to divide and expand clonally in the adult gland. In agreement with a recent report that intercalated duct cells harbor proliferative progenitor cells [27], genetic tracing in *Dcpp1*^*GCE/+*^*/ R26*^*TdT/+*^ mice for 3 months showed evidence of duct cell expansion within the intercalated ducts of the parotid gland. However, there was little evidence of acinar cell replenishment from the labeled intercalated duct cells. This suggests that acinar cells are not generally replaced by stem cells located within the intercalated ducts. Taken together, cell tracing using the *Pip*^*GCE*^ and *Dcpp1*^*GCE*^ strains supports a revised model of acinar cell homeostasis [28], which does not strictly depend on stem cells.

The reason for the unexpected ectopic expression of the Tcf^GCE^ in acinar cells is not clear. In a previously published report, insertion of the βgeo gene trap construct into Exon 2 of the *Tcfcp2l1* gene showed expression that was limited to duct cells [18]. In our targeting construct, the GCE cassette was inserted into Exon 1 at the initiation site (Fig 4A), suggesting that downstream sequences may be required for duct cell-specific expression. Although activation of the reporter in both duct and acinar cells by Tcf^GCE^ could be interpreted as evidence of a stem cell, we consider this unlikely. First, the ectopic labeling of acinar cells is detected within 24 hours of tamoxifen administration, a short time window for tracing of acinar cells putatively derived from a ductal stem cell. Second, in comparison to one day (Fig 4B and 4D), gavage on 3 consecutive days resulted in extensive acinar cell labeling (Fig 4C and 4E), suggesting that increased numbers of labeled acinar cells is dependent on tamoxifen dose. Third, the high number of labeled acinar cells generated after the short 3-day chase is inconsistent with recent reports demonstrating a lack of significant duct cell contribution to acinar cell replacement under normal homeostatic conditions [7, 27].

We anticipate that the Cre driver strains described here will facilitate numerous types of investigations into salivary gland biology, including a more detailed analysis of each specific cell type, to provide further insight into their lineage relationships. These alleles can also be used to analyze knockout phenotypes as the GCE cassette has been inserted at the start codon to yield a null allele in all three genes. Recently, it was discovered that Tcfcp2l1 plays a central role in sustaining ES cell pluripotency through the LIF/Stat3 signaling pathway [29, 30]. The Tcfcp2l1 transcription factor can reprogram post-implantation epiblast stem cells to ES cells. Although we have not investigated this, the *Tcf*^*GCE*^ allele might prove useful for early embryonic studies. In humans, PIP has been used as a diagnostic marker for breast cancer [31]. Although it has not yet been established whether the *Pip* gene is also activated in mouse breast cancer models, the availability of the *Pip*^*GCE*^ driver may provide a tool for such studies. Finally, these Cre drivers can also be used to target floxed genes, or to activate ectopic gene expression in a cell-specific manner. We expect that these alleles, which are freely available to the general scientific community, will be valuable tools for genetic manipulation in the salivary glands.

## Materials and Methods

### Generation of *GCE* knock-in mice

The *Pip*^*GCE*^ (MGI:5661584), *Tcfcp2l1*^*GCE*^ (MGI:5662395) and *Dcpp1*^*GCE*^ (MGI:5661581) mouse strains were produced in the Transgenic Facility at the University of Rochester. Genomic sequences for each targeted gene were isolated from Bac clones (ordered from Children’s Hospital Oakland Research Institute) by PCR amplification. The *Pip*^*GCE*^ targeting construct was generated by inserting the diphtheria toxin A gene (DTA, for positive selection), 4.4 kb 5’ homologous arm containing the 5’ UTR of Exon 1, and 4.7 kb 3’ homologous arm including Exon 2, 3 and 4 into pBluescript SKII(+). The eGFP-CreERT2 (GCE) fragment [19] with an SV40 polyadenylation site and Neo cassette were inserted immediately downstream of the translational initiation codon ATG. The *Pip*^*GCE*^ knock-in construct removed the coding sequences from Exon 1 and placed the GCE gene under the control of *Pip* regulatory sequences.

The *Tcfcp2l1*^*GCE*^ and *Dcpp1*^*GCE*^ targeting constructs were generated in a similar manner. Briefly, a 3.2 kb 5’ homologous arm containing the 5’ UTR of Exon 1 and 5.5 kb 3’ homologous arm containing Exon 2 were used for the *Tcfcp2l1*^*GCE*^ targeting construct. A 3 kb 5’ homologous arm containing the 5’ UTR of Exon 1, and 6.9 kb 3’ homologous arm including Exons 2, 3 and 4 were used for the *Dcpp1*^*GCE*^ targeting construct. Both *GCE* knock-in constructs removed the coding sequences downstream from the initiation codon and placed the GCE gene under the respective regulatory sequences.

To generate *Pip*^*GCE*^ knock-in mice, the *Pip*^*GCE*^ targeting construct was linearized with *NotI* and was electroporated into 129S6-C57BL/6J embryonic stem (ES) cells. Two targeted ES clones were identified by Southern blotting using an external 3’ probe (Fig 2A, gray box) and were injected into C57BL/6J blastocysts to generate mouse chimeras. Chimeras were mated with C57BL/6J mice to generate heterozygous *Pip^GCE/+^* mice. PCR method was used to genotype mice generated from subsequent breeding of *Pip^GCE/+^* heterozygotes. The PCR primers used to identify the GCE knock-in allele are An3’: 5- CCA CAC CTC CCC CTG AAC CTG -3’ and *Pip*R: 5’- GCT CTC ATT CTC AGA GAC TCC TG -3’.

To generate *Dcpp1*^*GCE*^ knock-in mice, the *Dcpp1*^*GCE*^ targeting construct (Fig 3A) was linearized with Asc*I* and was electroporated into 129S6-C57BL/6J embryonic stem (ES) cells. Nine correctly targeted ES clones were identified by 5’ long range PCR using an external 5’ primer and GCE internal primer; two of the clones were injected into C57BL/6J blastocysts to generate mouse chimeras. Chimeras were mated with C57BL/6J mice to generate heterozygous *Dcpp1^GCE/+^* mice. The long range PCR primers used to identify ES clones are: 5’ external primer DCPP1 F 11948–86: 5’- GCA GAC AGC CAA CAA GTA CTC TTC GAC TCC TGA CCT TG -3’ and GCE internal primer GCEmer308-269R: 5’-GAA GTC GTG CTG CTT CAT GTG GTC GGG GTA GCG GCT GAA G -3’; primers used to identify the GCE knock-in allele are An3’: 5’- CCA CAC CTC CCC CTG AAC CTG -3’ and *Dcpp1*R: 5’- GCA GAC TTC AGA CTT GAC TTA CCT TGT GTC -3’.

To generate *Tcfcp2l1*^*GCE*^ knock-in mice, the *Tcfcp2l1*^*GCE*^ targeting construct was linearized with *AscI* and was electroporated into 129S6-C57BL/6J embryonic stem (ES) cells. Ten targeted ES clones were identified by Southern blotting using an external 5’ probe (Fig 4A, shaded box). Two were injected into C57BL/6J blastocysts to generate mouse chimeras. Chimeras were mated with C57BL/6J mice to generate heterozygous *Tcfcp2l1 ^GCE/+^* mice. The PCR primers used to identify the GCE knock-in allele are An3’: 5'- CCA CAC CTC CCC CTG AAC CTG -3’ and *Tcfcp*R: 5’- TGC AGC GCA GAC CTG CT -3’.

The neomycin gene cassette was removed from each targeted allele by crossing with the Actin-Flippase mouse strain (Jackson Laboratory) [32]. All three strains were subsequently backcrossed onto a C57Bl/6 background. Mice were maintained on a 12-hour light/dark cycle in a one-way, pathogen-free facility at the University of Rochester Medical Center. Food and water were provided *ad libitum*.

Characterization of GCE expression in each strain was done using the R26-CMV-TdTomato (*R26*^*TdT/ TdT*^) reporter mouse strain purchased from Jackson Laboratory (Bar Harbor, Maine). Cre-positive males were mated with *R26*^*TdT/TdT*^ females to generate double heterozygotes. Genotyping for TdTomato-positive double heterozygotes used the following PCR primers: Cre: forward 5’- CACGACCAAGTGACAGCAATGCT- 3’, reverse 5’- CCATCGCTCGACCAGTTTAGTTACC- 3’; RTR: forward 5’- CTGTTCCTGTACGGCATGG- 3’, reverse 5’- GGCATTAAAGCAGCGTATCC- 3’. R26^TdT/ +^ heterozygotes were used as negative controls. Tissues were obtained from mice after euthanasia with CO_2_, followed by a secondary method (cervical dislocation).

This study was carried out in strict accordance with the recommendations in the Guide for the Care and Use of Laboratory Animals of the National Institutes of Health. The protocol was approved by the University Committee on Animal Resources at the University of Rochester Medical Center (protocol: 101362).

### Tamoxifen administration

Tamoxifen (156738, MP Biomedicals) was dissolved at 20 mg/ml or 40 mg/ml in corn oil (Sigma) and administered by intraperitoneal injection (i.p.) at 0.075 mg/g body weight, or by oral gavage at 0.25 mg/g body weight (adults and neonatal pups) on 3 consecutive days. To induce Cre in embryos, 0.125 mg/g body weight of tamoxifen was administered to the pregnant female by i.p. for 1 or 2 consecutive days. Tissues were harvested 3 days after tamoxifen administration unless otherwise indicated.

### Histology

Tissues were harvested and fixed 30 min on ice in 2% paraformaldehyde, 0.25% glutaraldehyde, 0.01% IGEPAL in PBS after 3 days or 3 month (for long-term lineage tracing) tamoxifen administration. The fixed tissues were rinsed in PBS, equilibrated overnight in 30% sucrose and embedded into OCT compound (Tissue-Tek). Sections (10 *μ*m thickness) were stained by DAPI (10 *μ*g/mg) and observed under the fluorescent microscope (IX81, Olympus) or confocal microscope (TCS SP5, Leica microsystems). For immunohistochemical analysis, tissues were fixed overnight in 4% paraformaldehyde in PBS at 4 degree, embedded into paraffin and cut into 5 *μ*m sections. All sections were deparaffinized and subjected to antigen retrieval in sodium citrate buffer (10 mM sodium citrate, 0.05% Tween20, pH6.0) for 10 min with heating. Other steps were performed as described previously (Bullard et al., 2008). Primary antibodies used were rabbit anti-RTR (1: 500, Rockland) and goat polyclonal anti-Nkcc1 antibody (1:100, sc-21545, Santa Cruz Biotechnology), rabbit polyclonal anti-Dcpp (Gift from Dr. Art R. Hand, University of Connecticut Health Center), and rabbit anti-Pip antibody (generous gift from Dr. Yvonne Myal, University of Manitoba) ([8], 1: 200). Secondary antibodies used were Cy3-conjugated donkey anti-rabbit or Cy2 labeled donkey anti-goat antibody (1: 500, Jackson ImmunoResearch).

### Imaging

Imaging was done using an Olympus iX81 microscope, Hamamatsu CCD camera, and MetaMorph software. Adobe Illustrator® CS6 and Photoshop® CS5 (Adobe Systems Incorporated, San Jose, CA) were used to compile illustrations and to perform image adjustments. All changes in contrast and brightness were applied to the entire image.

## Supporting Information

S1 Fig Pip expression in adult salivary and lacrimal glands. Upper panel: Antibody to Pip was used for immunohistochemistry on sections of submandibular (SMG), sublingual (SLG), parotid (Par), and lacrimal gland (Lac). Positive cells are brown due to labeling with DAB. White arrows indicate serous demilune cells in SLG. Lower panel was treated with secondary antibody only. Scale bars = 100 *μ*m
(TIF)

S2 Fig Endogenous Dcpp1 expression in sublingual and parotid glands. Antibody to Dcpp1 (generous donation from Dr. Art Hand, University of Connecticut School of Dentistry) highlights endogenous expression pattern on sections of **(A)** serous demilune cells of the SLG (arrowhead); and **(B)** intercalated ducts of the parotid gland (arrow). Acini are outlined. Scale bars = 20 *μ*m.
(TIF)

## References

[1] Pinkstaff C. Biology of the salivary glands University of Michigan: CRC Press; 1993.

[2] Fox PC. Acquired salivary dysfunction. Drugs and radiation. Ann N Y Acad Sci. 1998;842:132–7. Epub 1998/05/26. . 9599303 10.1111/j.1749-6632.1998.tb09641.x

[3] Lombaert IM, Brunsting JF, Wierenga PK, Faber H, Stokman MA, Kok T, et al Rescue of salivary gland function after stem cell transplantation in irradiated glands. PLoS One. 2008;3(4):e2063 Epub 2008/05/01. 10.1371/journal.pone.0002063 18446241 PMC2329592

[4] Nanduri LS, Lombaert IM, van der Zwaag M, Faber H, Brunsting JF, van Os RP, et al Salisphere derived c-Kit+ cell transplantation restores tissue homeostasis in irradiated salivary gland. Radiother Oncol. 2013;108(3):458–63. Epub 2013/06/19. 10.1016/j.radonc.2013.05.020 . 23769181

[5] Xiao N, Lin Y, Cao H, Sirjani D, Giaccia AJ, Koong AC, et al Neurotrophic factor GDNF promotes survival of salivary stem cells. J Clin Invest. 2014;124(8):3364–77. Epub 2014/07/19. 10.1172/JCI74096 25036711 PMC4109543

[6] Pringle S, Van Os R, Coppes RP. Concise review: Adult salivary gland stem cells and a potential therapy for xerostomia. Stem Cells. 2013;31(4):613–9. Epub 2013/01/22. 10.1002/stem.1327 . 23335219

[7] Aure MH, Konieczny SF, Ovitt CE. Salivary gland homeostasis is maintained through acinar cell self-duplication. Dev Cell. 2015;33(2):231–7. Epub 2015/04/07. 10.1016/j.devcel.2015.02.013 25843887 PMC4406828

[8] Blanchard A, Nistor A, Castaneda FE, Martin D, Hicks GG, Amara F, et al Generation and initial characterization of the prolactin-inducible protein (PIP) null mouse: accompanying global changes in gene expression in the submandibular gland. Can J Physiol Pharmacol. 2009;87(10):859–72. Epub 2010/01/07. 10.1139/Y09-077 . 20052012

[9] Murphy LC, Tsuyuki D, Myal Y, Shiu RP. Isolation and sequencing of a cDNA clone for a prolactin-inducible protein (PIP). Regulation of PIP gene expression in the human breast cancer cell line, T-47D. J Biol Chem. 1987;262(31):15236–41. Epub 1987/11/05. . 3667631

[10] Myal Y, Iwasiow B, Yarmill A, Harrison E, Paterson JA, Shiu RP. Tissue-specific androgen-inhibited gene expression of a submaxillary gland protein, a rodent homolog of the human prolactin-inducible protein/GCDFP-15 gene. Endocrinology. 1994;135(4):1605–10. Epub 1994/10/01. 10.1210/endo.135.4.7925123 . 7925123

[11] Bekhor I, Wen Y, Shi S, Hsieh CH, Denny PA, Denny PC. cDNA cloning, sequencing and in situ localization of a transcript specific to both sublingual demilune cells and parotid intercalated duct cells in mouse salivary glands. Arch Oral Biol. 1994;39(12):1011–22. Epub 1994/12/01. . 7717881 10.1016/0003-9969(94)90052-3

[12] Mullins JJ, Mullins LJ, Dunbar DR, Brammar WJ, Gross KW, Morley SD. Identification of a human ortholog of the mouse Dcpp gene locus, encoding a novel member of the CSP-1/Dcpp salivary protein family. Physiol Genomics. 2006;28(1):129–40. Epub 2006/09/07. 10.1152/physiolgenomics.00153.2006 . 16954406

[13] Redman RS, Ball WD. Cytodifferentiation of secretory cells in the sublingual gland of the prenatal rat: a histological, histochemical and ultrastructural study. Am J Anat. 1978;153(3):367–89. Epub 1978/11/01. 10.1002/aja.1001530304 . 707321

[14] Ball WD, Hand AR, Johnson AO. Secretory proteins as markers for cellular phenotypes in rat salivary glands. Dev Biol. 1988;125(2):265–79. Epub 1988/02/01. . 2828136 10.1016/0012-1606(88)90210-2

[15] Ball WD, Hand AR, Moreira JE, Johnson AO. A secretory protein restricted to type I cells in neonatal rat submandibular glands. Dev Biol. 1988;129(2):464–75. Epub 1988/10/01. . 3046963 10.1016/0012-1606(88)90393-4

[16] Arnold K, Sarkar A, Yram MA, Polo JM, Bronson R, Sengupta S, et al Sox2(+) adult stem and progenitor cells are important for tissue regeneration and survival of mice. Cell Stem Cell. 2011;9(4):317–29. Epub 2011/10/11. 10.1016/j.stem.2011.09.001 21982232 PMC3538360

[17] Rodda S, Sharma S, Scherer M, Chapman G, Rathjen P. CRTR-1, a developmentally regulated transcriptional repressor related to the CP2 family of transcription factors. J Biol Chem. 2001;276(5):3324–32. Epub 2000/11/14. 10.1074/jbc.M008167200 . 11073954

[18] Yamaguchi Y, Yonemura S, Takada S. Grainyhead-related transcription factor is required for duct maturation in the salivary gland and the kidney of the mouse. Development. 2006;133(23):4737–48. Epub 2006/11/03. 10.1242/dev.02658 . 17079272

[19] Mugford JW, Sipila P, Kobayashi A, Behringer RR, McMahon AP. Hoxd11 specifies a program of metanephric kidney development within the intermediate mesoderm of the mouse embryo. Dev Biol. 2008;319(2):396–405. Epub 2008/05/20. 10.1016/j.ydbio.2008.03.044 18485340 PMC2580739

[20] Mirels L, Hand AR, Branin HJ. Expression of gross cystic disease fluid protein-15/Prolactin-inducible protein in rat salivary glands. J Histochem Cytochem. 1998;46(9):1061–71. Epub 1998/08/26. . 9705972 10.1177/002215549804600910

[21] Lee B, Modha G, Watson PH, Dodd J, Troup S, Blanchard A, et al Expression of the mouse homologue for the human GCDFP-15/PIP gene during pre- and early post-natal development. Mol Cell Endocrinol. 2003;205(1–2):33–41. Epub 2003/08/02. . 12890565 10.1016/s0303-7207(03)00210-7

[22] Mazoujian G, Pinkus GS, Davis S, Haagensen DE Jr. Immunohistochemistry of a gross cystic disease fluid protein (GCDFP-15) of the breast. A marker of apocrine epithelium and breast carcinomas with apocrine features. Am J Pathol. 1983;110(2):105–12. Epub 1983/02/01. 6130702 PMC1916150

[23] Denny PC, Denny PA. Dynamics of parenchymal cell division, differentiation, and apoptosis in the young adult female mouse submandibular gland. Anat Rec. 1999;254(3):408–17. Epub 1999/03/30. . 10096673 10.1002/(SICI)1097-0185(19990301)254:3<408::AID-AR12>3.0.CO;2-G

[24] Man YG, Ball WD, Marchetti L, Hand AR. Contributions of intercalated duct cells to the normal parenchyma of submandibular glands of adult rats. Anat Rec. 2001;263(2):202–14. Epub 2001/05/22. . 11360236 10.1002/ar.1098

[25] Schwartz-Arad D, Arber L, Arber N, Zajicek G, Michaeli Y. The rat parotid gland—a renewing cell population. J Anat. 1988;161:143–51. Epub 1988/12/01. 3254887 PMC1262098

[26] Zajicek G, Yagil C, Michaeli Y. The streaming submandibular gland. Anat Rec. 1985;213(2):150–8. Epub 1985/10/01. 10.1002/ar.1092130206 . 4073568

[27] Kwak M, Ghazizadeh S. Analysis of histone H2BGFP retention in mouse submandibular gland reveals actively dividing stem cell populations. Stem Cells Dev. 2015;24(5):565–74. Epub 2014/09/23. 10.1089/scd.2014.0355 25244667 PMC4333511

[28] Aure MH, Arany S, Ovitt CE. Salivary Glands: Stem Cells, Self-duplication, or Both? J Dent Res. 2015 Epub 2015/08/20. 10.1177/0022034515599770 PMC4622322 26285812

[29] Martello G, Bertone P, Smith A. Identification of the missing pluripotency mediator downstream of leukaemia inhibitory factor. EMBO J. 2013;32(19):2561–74. Epub 2013/08/15. 10.1038/emboj.2013.177 23942233 PMC3791366

[30] Ye S, Li P, Tong C, Ying QL. Embryonic stem cell self-renewal pathways converge on the transcription factor Tfcp2l1. EMBO J. 2013;32(19):2548–60. Epub 2013/08/15. 10.1038/emboj.2013.175 23942238 PMC3791365

[31] Mazoujian G, Bodian C, Haagensen DE Jr., Haagensen CD. Expression of GCDFP-15 in breast carcinomas. Relationship to pathologic and clinical factors. Cancer. 1989;63(11):2156–61. Epub 1989/06/01. . 2655863 10.1002/1097-0142(19890601)63:11<2156::aid-cncr2820631115>3.0.co;2-b

[32] Rodriguez CI, Buchholz F, Galloway J, Sequerra R, Kasper J, Ayala R, et al High-efficiency deleter mice show that FLPe is an alternative to Cre-loxP. Nat Genet. 2000;25(2):139–40. Epub 2000/06/03. 10.1038/75973 . 10835623

