# Supplementary figures and images for: Cell-Specific Cre Strains For Genetic Manipulation in Salivary Glands
Source: PLoS One. 2016 Jan 11;11(1):e0146711. doi: 10.1371/journal.pone.0146711 (PMC4709230; doi:10.1371/journal.pone.0146711)

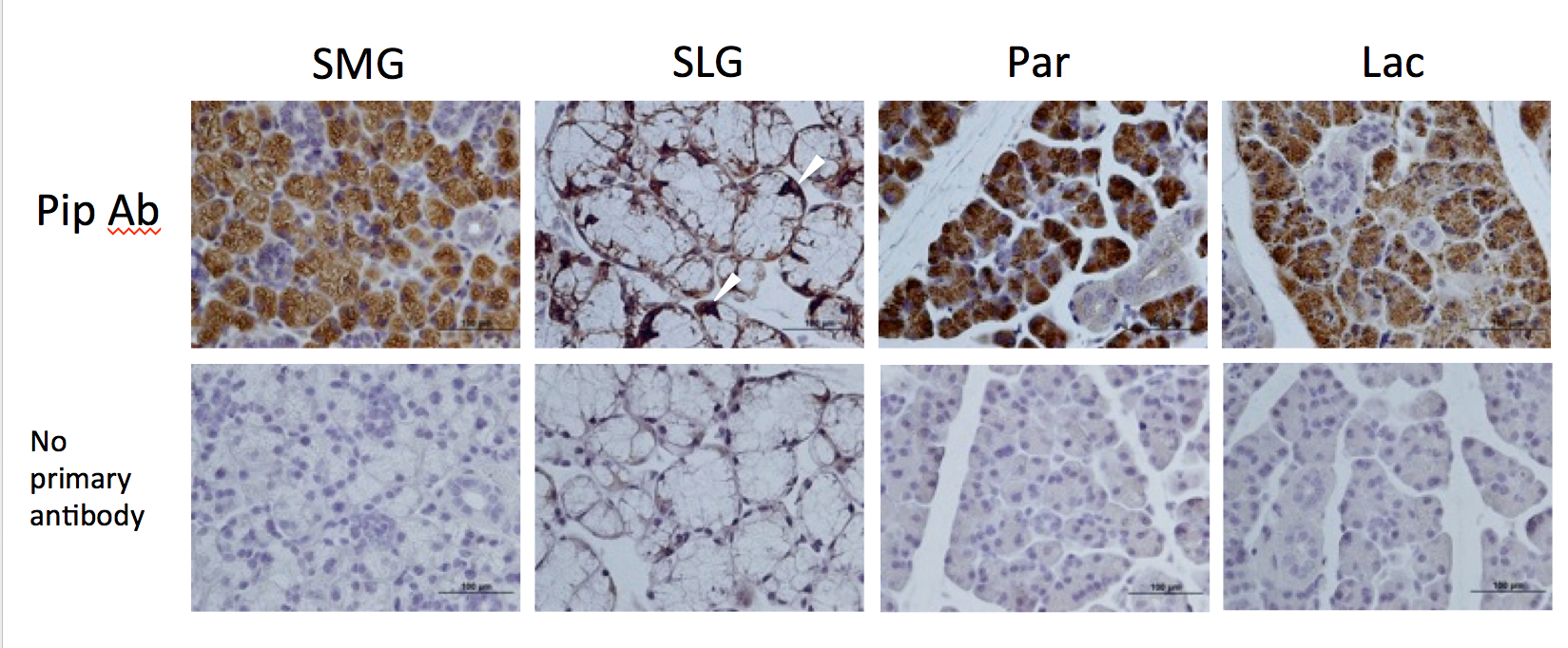

Supplement: S1 Fig — Upper panel: Antibody to Pip was used for immunohistochemistry on sections of submandibular (SMG), sublingual (SLG), parotid (Par), and lacrimal gland (Lac). Positive cells are brown due to labeling with DAB. White arrows indicate serous demilune cells in SLG. Lower panel was treated with secondary antibody only. Scale bars = 100 μm (TIF) [file pone.0146711.s001.tif]

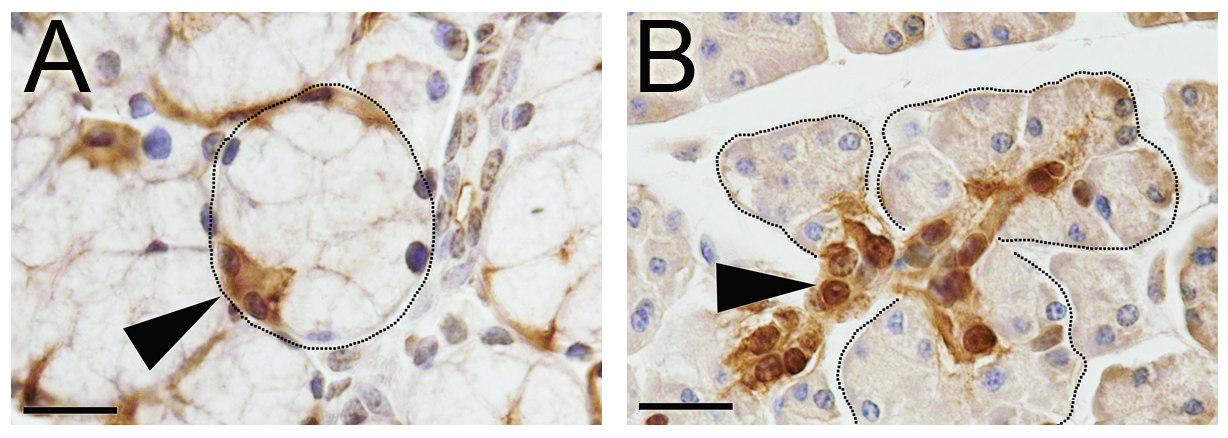

Supplement: S2 Fig — Antibody to Dcpp1 (generous donation from Dr. Art Hand, University of Connecticut School of Dentistry) highlights endogenous expression pattern on sections of (A) serous demilune cells of the SLG (arrowhead); and (B) intercalated ducts of the parotid gland (arrow). Acini are outlined. Scale bars = 20 μm. (TIF) [file pone.0146711.s002.tif]
